# Humans, Animals, Food and Environment: One Health Approach against Global Antimicrobial Resistance

**DOI:** 10.3390/antibiotics9060346

**Published:** 2020-06-19

**Authors:** Marcello Iriti, Sara Vitalini, Elena Maria Varoni

**Affiliations:** 1 Department of Agricultural and Environmental Sciences, Milan State University, via G. Celoria 2, 20133 Milan, Italy; sara.vitalini@unimi.it; 2 Department of Biomedical, Surgical and Dental Sciences, Milan State University, via Beldiletto 1/3, 20122 Milan, Italy; elena.varoni@unimi.it

**Keywords:** antibiotics, infectious diseases, multi-drug resistance, natural products, plant extracts, phytochemicals

## Abstract

Antimicrobial resistance represents one of the most relevant threats to global public health and food security, affecting anyone, of any age, in any country and is responsible for longer hospital stays, higher medical costs and increased mortality. Resistant microorganisms are present in humans, animals, food and the environment, and, therefore, the One Health approach is very promising to counteract antimicrobial resistance, since human health and animal health are connected to each other and with the environment and the latter a potential source of resistant microorganisms. In this context, the need for novel antimicrobial drugs has stimulated the exploration of plant products as a source of novel phytotherapeutics able to reverse antimicrobial resistance when used in combination with conventional antibiotic drugs.

The global burden of infectious diseases has been increasing in recent decades, mainly due to the phenomenon of antimicrobial resistance (Table 1), one of the most demanding global challenges from the beginning of the Antibiotic Era, product of Paul Ehrlich and Alexander Fleming. A number of factors exacerbate antimicrobial resistance, in addition to the misuse and overuse of antibiotics (the major drivers), including a lack of access to quality primary health care and poverty, as well as inadequate water, sanitation and hygiene.

Inequities in healthcare and drug access represent another relevant issue in many low- and middle-income countries that continue to be burdened with high rates of infectious diseases and low rates of antibiotic use. The main resistant bacteria that commonly cause infections in hospitals and communities are reported in Table 2 [5].

Global antibiotic consumption in humans increased by 36% between 2000 and 2010, with only five countries (Brazil, Russia, India, China and South Africa) and two antibiotic classes (cephalosporins and broad-spectrum penicillins) accounting for 76% and 55% of the increase, respectively. It is noteworthy that the consumption of two last-resort classes of antibiotics, carbapenems and polymixins, also increased by 45% and 13%, respectively [1]. If no effective action is promptly put in place, the projected global antibiotic use in 2030 is estimated to dramatically increase by up to 200% compared to that in 2015, with the consumption rate in low- and middle-income countries reaching (and in some countries surpassing) the levels of high income countries [2]. Not least, globalization facilitates the spread of antimicrobial resistance due to increased trade, travel and human and animal migrations. Indeed, most antimicrobials are consumed by animals, not by man, as is commonly believed. In the US, the livestock sector accounts for about 80% of the total annual consumption. Between 2010 and 2030, the global consumption of antimicrobials in this sector has been projected to increase by about 67% [3].

In the EU, the use of antibiotics for the treatment of multidrug-resistant bacterial infections doubled in 2010–2014 (Figure 1).

Each year, more than 670,000 infections due to resistant bacteria occur in the EU and 33,000 people die as a direct consequence of these infections (Figure 2). For instance, between 2007 and 2015, the number of deaths attributable to infections with *Klebsiella pneumoniae* resistant to carbapenems (a group of last-line antibiotics) and with third generation cephalosporin-resistant *Escherichia coli* increased six-fold and four-fold, respectively [4]. In 2018, resistance to ampicillin, sulfonamides and tetracyclines was recorded in *Salmonella* spp. from human cases, particularly among serovars commonly found in pigs [6]. Indeed, according to the European Medicines Agency (EMA), in 2017, the best-selling antimicrobial veterinary medicinal products in the EU were tetracyclines (30.4%), penicillins (26.9%) and sulfonamides (9.2%) [7]. Assuming there is no policy change and antimicrobial resistance rates follow the projected trends, up to 1.1 billion euros are expected to be spent yearly, between 2015 and 2050, due to antimicrobial resistance, in the EU [4].

The history of antimicrobial discovery is quite curious, until the 1970s, many new antibacterial drugs were developed, but the most recent completely new classes of antibacterial drugs were discovered during the 1980s. In other words, a “discovery void” exists, i.e., over the last 30 years, no major new type of antibiotic has been developed (Table 3) [8].

In this view, the urgent need for novel antimicrobial drugs to reduce the global burden of infectious diseases has greatly stimulated the exploration of plant products as a source of novel and effective phytotherapeutic agents. A plethora of preclinical studies demonstrated the efficacy of plant aqueous and solvent extracts, essential oils and isolated phytochemicals in the control of pathogenic microorganisms, including bacterial- and fungal-resistant strains (Table 4).

In this complex scenario, it is evident that resistant microorganisms are present in humans, animals, food and the environment. Therefore, the One Health (or superorganism) approach is very promising against antimicrobial resistance, since human and animal health are interconnected, in the sense that diseases are transmitted from humans to animals and vice versa. The environment represents another link between humans and animals, and, at the same time, a potential source of new resistant microorganisms. In 2017, the EU launched the European One Health Action Plan against Antimicrobial Resistance with the aims of supporting the member states in reducing the emergence and spread of antimicrobial resistance and increasing the development and availability of new effective antimicrobials inside and outside the EU. This plan covers the full One Health spectrum, addressing human health and animal health, as well as the role of the environment, providing a framework of actions in order to boost research, development and innovation in antimicrobial resistance: (1) improve knowledge of detection, effective infection control and surveillance; (2) develop new therapeutics and alternatives; (3) develop new preventive vaccines; (4) develop novel diagnostics; (5) develop new economic models and incentives; (6) close knowledge gaps on antimicrobial resistance in the environment and on how to prevent transmission. These actions strengthen collaboration and surveillance, creating more synergies and partnerships for stronger cooperation with developing countries [16].

The EU action plan certainly strengthens technical cooperation with the WHO Global Action Plan on Antimicrobial Resistance, whose goal is to ensure, for as long as possible, the continuity of successful treatment and prevention of infectious diseases with effective and safe medicines that are quality assured, used in a responsible way and accessible to all who need them. To achieve this goal, the WHO action plan sets out five strategic objectives: (1) to improve the awareness and understanding of antimicrobial resistance through effective communication, education and training; (2) to strengthen knowledge and evidence bases through surveillance and research; (3) to reduce the incidence of infection through effective sanitation, hygiene and infection prevention measures; (4) to optimize the use of antimicrobial agents in human and animal health; (5) to ensure sustainable investment (in new medicines, diagnostic tools, vaccines and other interventions) in countering antimicrobial resistance that takes account of the needs of all countries [17].

At the end of this brief commentary, it appears evident that the One Health archetype represents the right path to counteract antimicrobial resistance, a multifactorial phenomenon requiring a structured and diversified control strategy. In this context, plant products represent a nearly unlimited source of (multitarget) active ingredients, consisting of complex mixtures of hundreds of different compounds that may be synergistically active once administered. In addition, plant extracts and phytochemicals can be useful in adjuvant therapy to improve the efficacy of conventional antimicrobials (Table 4), to decrease their adverse effects and to reverse multidrug resistance, the latter an emerging and very critical topic due to the genetic plasticity and environmental adaptability of pathogenic microorganisms [18]. Not least, selected phytochemicals can also be used as a template (“lead compounds”) for the development of new chemical scaffolds in modern drug discovery—nature still remains the largest organic chemistry laboratory in the world.

## Figures and Tables

**Figure 1:**
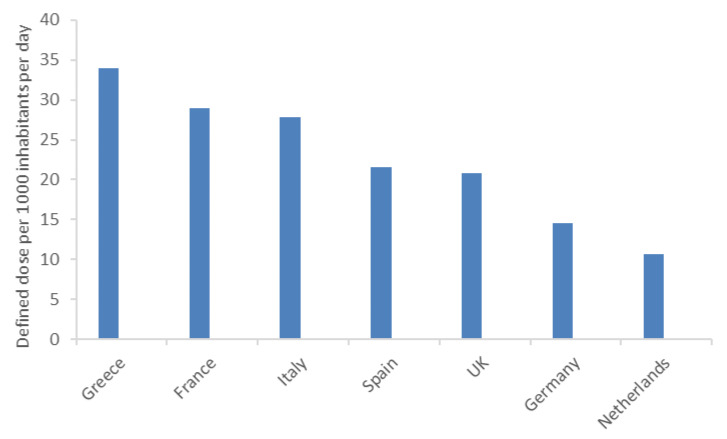
Antibiotic consumption (defined as dose per 1000 inhabitants per day) across EU countries in 2014. Adapted from [4].

**Figure 2:**
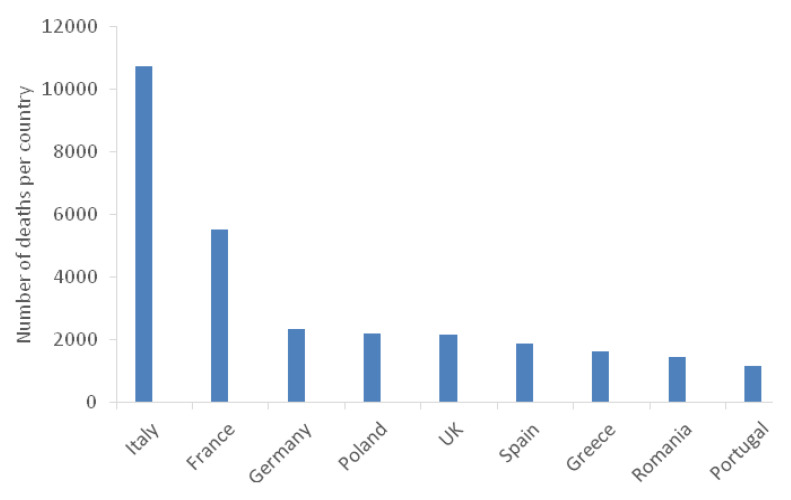
Number of deaths caused by antibiotic-resistant bacteria by EU country. Adapted from [4].

**Table 1:** Antimicrobial resistance, a major global threat: factsheet.

Global antibiotic consumption in humans has increased by 36% between 2000 and 2010.	[1]
Projected global antibiotic use in 2030 is estimated to increase up to 200%, if no effective action is promptly put in place.	[2]
In the US, the livestock sector accounts for about 80% of total annual antibiotic consumption.	[3]
In the US, between 2010 and 2030, global consumption of antimicrobials in the livestock sector is projected to increase by about 67%.	[3]
In the EU, the use of antibiotics for the treatment of multidrug-resistant bacterial infections doubled in 2010–2014.	[4]
Each year, more than 670,000 infections due to resistant bacteria occur in the EU.	[4]
Each year, 33,000 people die, in the EU, as a direct consequence of these infections.	[4]
Up to 1.1 billion euros are expected to be spent yearly between 2015 and 2050 due to antimicrobial resistance in the EU, assuming there is no policy change and antimicrobial resistance rates follow the projected trends.	[4]

**Table 2:** Selected bacteria/resistance combinations.

Bacterium	Resistance/Decreased Susceptibility to
*Escherichia coli*	3rd generation cephalosporins, fluoroquinolones
*Klebsiella pneumoniae*	3rd generation cephalosporins, carbapenems
*Staphylococcus aureus*	Methicillin (β-lactam antibiotics)
*Streptococcus pneumoniae*	Penicillins
Nontyphoidal *Salmonella*	Fluoroquinolones
*Shigella* spp.	Fluoroquinolones
*Neisseria gonorrhoeae*	3rd generation cephalosporins

Adapted from [5].

**Table 3:** Dates of discovery of the classes of antibacterial drugs.

Antibacterial Drug	Year of Discovery
Salvarsan	1908
Penicillin	1928
Sulfonamide	1932
Streptomycin	1943
Bacitracin	1945
Nitrofurans	1946
Chloramphenicol, polymyxin	1947
Chlortetracycline, cephalosporin	1948
Pleuromutilin	1950
Erythromycin, isoniazid	1952
Vancomycin, streptogramin	1954
Cycloserine	1955
Novobiocin	1956
Rifamycin	1957
Metronidazole	1959
Nalidixic acid, trimethoprim, lincomycin, fusidic acid	1961
Fosfomycin	1969
Mupirocin	1971
Carbapenem	1976
Oxazolidinone	1978
Monobactam	1981
Daptomycin	1987
Discovery void	

Adapted from [8].

**Table 4:** Plant products with antimicrobial activity.

Plant Products	Target Organisms	Notes	References
*Prunus avium* tree bark methanol extracts	Seventeen Bacterial and five yeast strains	Antibiofilm activity against *Staphylococcus aureus*; dihydrowogonin was the most active constituent	[9]
*Mesosphaerum suaveolens* aqueous extracts (in combination with fluconazole)	*Candida* strains	The extract potentiated the antifungal drug	[10]
α-Bisabolol (in combination with tetracycline and norfloxacin)	*S. aureus* strains	The compounds potentiated the antibiotics inhibiting the bacterial efflux pumps	[11]
*Croton ceanothifolius* essential oil (EO) (in combination with penicillin, norfloxacin and gentamicin)	*S. aureus, Pseudomonas aeruginosa* and *Escherichia coli* multiresistant strains	The combination exhibited a synergistic effect	[12]
*Eugenia brasiliensis* and *Piper mosenii* EOs (in association with blue led light and aminoglycosides)	*S. aureus* and *E. coli* strains	The association of EOs with the blue LED light increased the activity of amikacin and gentamicin	[13]
*Spondias tuberosa* hydroalcoholic extracts (in combination with fluconazole)	*Candida* strains	Synergism with the fungicide	[14]
*Caulerpa racemosa* and *Caulerpa lentillifera* chloroform, methanol and aqueous extracts	Methicillin-resistant *S. aureus* and neuropathogenic *E. coli*	*Candida racemosa* chloroform extract showed the highest antibacterial activity	[15]
